# Pseudo-anévrisme post traumatique de la carotide externe

**DOI:** 10.11604/pamj.2017.28.272.11259

**Published:** 2017-11-28

**Authors:** Sadik Zbair, Jebrane Dinari, Abdellatif Siwane, Samira Lezar, Fatiha Essodegui

**Affiliations:** 1 Université Hassan II, Faculté de Médecine et de Pharmacie Casablanca, Département de Radiologie, Service Central de Radiologie, CHU Ibn Rochd, Casablanca, Maroc

**Keywords:** Carotide externe, pseudo-anévrisme, post traumatique, External carotid, pseudoaneurysm, post-traumatic

## Abstract

Le pseudo-anévrisme provient d'une solution de la continuité de la paroi artérielle, secondaire à l'inflammation, un traumatisme ou des causes iatrogènes telles que la chirurgie, une biopsie percutanée ou un drainage. Nous rapportons un cas d'un patient de 37 ans, venant consulter pour constatation d'une tuméfaction cervicale évoluant progressivement suite à un traumatisme cervical pénétrant survenu 8 mois auparavant. L'échographie doppler complétée par un angioscanner a confirmé le diagnostic de pseudo-anévrisme de l'artère carotide externe gauche. Le diagnostic de pseudo-anévrisme est plus aisé avec le développement considérable de l'imagerie en coupe et de l'angiographie.

## Abstract (en)

Pseudoaneurysm is due to a disruption in the arterial wall, secondary to inflammation, trauma or iatrogenic causes such as surgery, percutaneous biopsy or drainage. We report the case of a 37-year old patient, presenting with cervical swelling that had progressively evolved after penetrating cervical trauma occurred 8 months before. Doppler ultrasound supplemented by CT angiography confirmed the diagnosis of pseudoaneurysm of the left external carotid artery. The considerable development of sectional imaging techniques and of angiography has allowed easier diagnosis.

## Introduction

Les pseudo-anévrismes des artères de gros calibres et qui présentent des risques de complications graves sont rares dans les traumatismes cervico-faciaux. Nous rapportons un cas de traumatisme facial, survenu 8 mois auparavant, se présentant pour une masse cervicale antérieure gauche.

## Patient et observation

**Figure 1:**
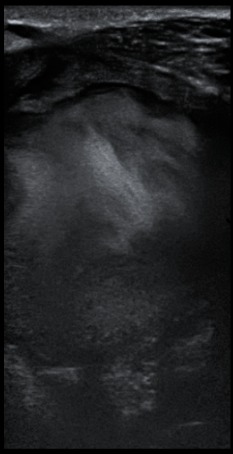
Échographie de la masse cervicale: formation arrondie siège de turbulence

**Figure 2:**
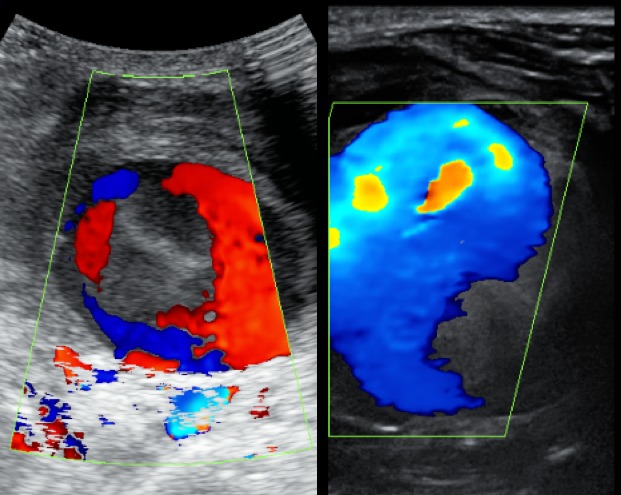
Turbulence au doppler couleur réalisant un aspect de Ying-Yang

**Figure 3:**
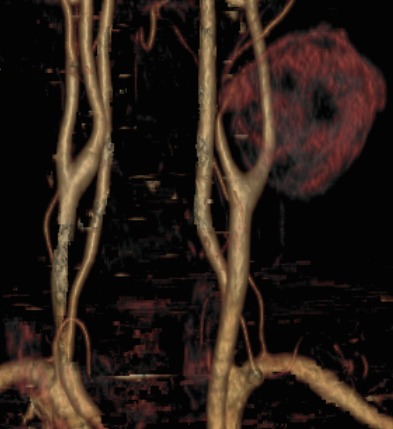
Angioscanner des TSA: reconstruction en VR montrant le pseudo-anévrisme de la carotide externe gauche

**Figure 4:**
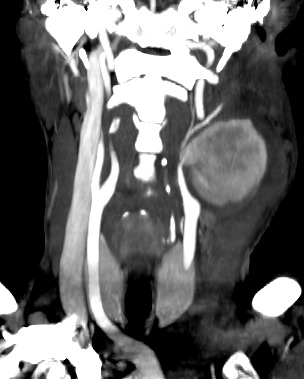
Angioscanner des TSA: coupe coronale MIP montrant le rehaussement hétérogène du pseudo-anévrisme au temps artériel

**Figure 5:**
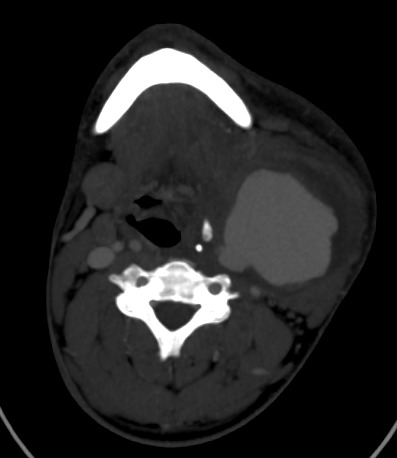
Angioscanner des TSA: coupe axiale montrant le rehaussement homogène du pseudo-anévrisme au temps veineux

**Figure 6:**
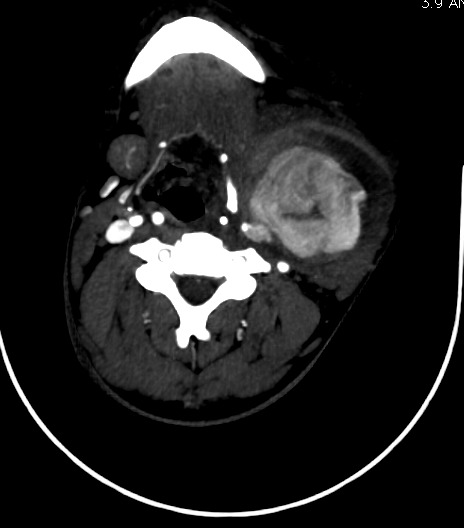
Angioscanner des TSA: coupe axiale montrant la présence d’un thrombus pariétal du pseudo-anévrisme

Un patient de sexe masculin, âgé de 37 ans vient consulter pour constatation d'une tuméfaction cervicale. A l'examen clinique on retrouve une masse cervicale sous maxillaire gauche, pulsatile avec auscultation d'un bruit audible en son sein sans signes inflammatoire en regard. L'anamnèse décèle une notion de traumatisme pénétrant par arme blanche survenu 8 mois auparavant avec des suites sans complications, puis l'évolution progressive de la masse sus décrite. Le patient a bénéficié d'une échographie avec étude doppler et complétée par un angioscanner des troncs supra-aortique (TSA). L'échographie a montré la présence d'une formation arrondie siège de turbulence, réalisant un aspect de Ying-Yang au doppler couleur (Figure 1, Figure 2). L'angioscanner a permis une étude plus étalée, en montrant une masse arrondie mesurant 6cm de diamètre appendue à la paroi artérielle de la carotide externe gauche (Figure 3), se rehaussant après injection de produit de contraste de façon hétérogène au temps artériel (turbulences) (Figure 4), s'homogénéisant à un temps plus tardif (Figure 5). On note également la présence d'un thrombus pariétal mesurant 16 mm d'épaisseur maximale (Figure 6). Le pseudo-anévrisme décrit un collet large mesurant 8 mm de calibre. Il s'y associe un effet de masse sur les parties molles et les axes vasculaires de voisinage avec infiltration de la graisse.

## Discussion

Un pseudo-anévrisme désigne une rupture focale d'une paroi artérielle avec maintien d'une communication avec son artère d'alimentation. Toutes les couches de la paroi artérielle (tunique externe, média et l'intima) doivent être rompues. Le pseudo-anévrisme peut être secondaire a de nombreuses étiologies dont les plus courantes sont traumatiques, iatrogènes et inflammatoires. L''avènement de l'imagerie moderne a rendu le diagnostic de pseudo-anévrisme plus fréquent [1,2]. Les pseudo-anévrismes sont souvent asymptomatiques et de découverte fortuite. Lorsqu'ils sont symptomatiques, les manifestations locales sont habituellement secondaires à leur effet de masse. Elles se traduisent par une sensation de thrill local, un bruit audible ou par une masse pulsatile, quelques fois de l''dème par compression des structures veineuses adjacentes [3]. La thrombose et l'infection du pseudo-anévrisme peuvent également se produire. La rupture est une complication particulièrement redoutée car elle peut entraîner une hémorragie et un choc hypovolémique. Il existe plusieurs modalités d'imagerie pour l'exploration des pseudo-anévrismes; l'échographie en mode B montre une structure kystique qui se continue avec l'artère d'alimentation par un collet. L'échographie peut également donner une évaluation interne du pseudo-anévrisme, qui peut démontrer des septas, un thrombus. Le mode Doppler montre fréquemment un signe de Ying-Yang, qui décrit un modèle de flux sanguin tourbillonnant dans le sac. En outre, le Doppler pulsé peut démontrer la communication du pseudo-anévrisme avec l'artère d'alimentation, produisant une forme d'onde artérielle "à va-et-vient", représentée par le sang s'écoulant dans (pendant la systole) et hors de (pendant la diastole) le collet du pseudo-anévrisme [2,4]. Sur un scanner non injecté, le pseudo-anévrisme peut être vu comme une formation ronde hypodense accolée à l'artère d'intérêt associée à une infiltration des structures adjacentes de densité intermédiaire ou élevée selon la chronicité. Une apparence similaire sera observée sur l'examen avec injection de produit de contraste (PDC); le sac pseudo anévrismal partiellement ou complètement rempli par le PDC, en fonction de la présence possible d'une thrombose. Les pseudo-anévrismes sont généralement bien délimités avec des parois fines, à l'exception des pseudo-anévrismes mycotiques ayant tendance à avoir des parois plus irrégulières et épaissies [3]. Chez les patients où l'angioscanner ne peut être réalisé ou est contre indiqué en raison de la fonction rénale altérée ou de l'allergie au produit de contraste, l'angiographie par résonnance magnétique (ARM) est une alternative. L'angiographie conventionnelle bénéficie d'une excellente résolution spatiale et permet une étude dynamique des troncs supra aortiques, et de la collatéralité. Elle est toujours la technique de référence pour la détection des pseudo-anévrismes. Grâce aux acquisitions en 3 dimensions, elle permet une caractérisation très précise de la morphologie du pseudo-anévrisme, de sa forme, de ses contours et de leur régularité, une mesure de la taille de la poche et surtout des dimensions du collet et de l'anatomie de l'artère d'alimentation. Elle permet également d'étaler la gamme des diagnostics différentiels (par exemple, les fistules et les malformations vasculaires). Cependant, l'angiographie conventionnelle est un examen invasif irradiant pour le patient et le médecin. C'est la raison pour laquelle cet examen n'est actuellement pas réalisé pour le diagnostic positif de pseudo-anévrisme, mais pour la planification thérapeutique, voire uniquement réalisée lors du traitement end vasculaire. Le traitement des pseudo-anévrismes symptomatiques peut être réalisé par plusieurs méthodes incluant la surveillance devant la possibilité de thrombose spontanée concernant les pseudo-anévrismes de petite taille, la thrombose pharmacologique avec compression guidée par l'échographie, la chirurgie (utilisant le pontage ou la ligature), le stenting et l'embolisation [5]. La chirurgie est indiquée quand il existe des preuves d'une infection mycotique surajoutée, une ischémie distale, un déficit neurologique. Cependant, le protocole de traitement n'est pas universel, et la gestion tient compte de la particularité de chaque présentation clinique [6].

## Conclusion

Le diagnostic du pseudo-anévrisme est devenu plus aisé avec l'avènement des nouvelles technologies de l'imagerie en coupe et de l'angiographie, en permettant un diagnostic plus précoce et concis. Afin d'assurer une prise en charge urgente et éviter des complications fatales pour le malade.

## Conflits d’intérêts

Les auteurs ne déclarent aucun conflit d'intérêts.
